# Can We Learn to Treat One Another Better? A Test of a Social Intelligence Curriculum

**DOI:** 10.1371/journal.pone.0128638

**Published:** 2015-06-15

**Authors:** Eva K. Zautra, Alex J. Zautra, Carmen Ecija Gallardo, Lilian Velasco

**Affiliations:** 1 Social Intelligence Institute, Phoenix, Arizona, United States of America; 2 Department of Psychology, Arizona State University, Tempe, Arizona, United States of America; 3 Department of Psychology, Universidad Rey Juan Carlos, Madrid, Spain; University of Portsmouth, UNITED KINGDOM

## Abstract

This paper reports on the first test of the value of an online curriculum in social intelligence (SI). Built from current social and cognitive neuroscience research findings, the 50 session SI program was administered, with facilitation in Spanish by classroom instructors, to 207 students from Universidad Rey Juan Carlos in Madrid as part of their undergraduate classes. All materials were translated into Castilian Spanish, including outcome measures of SI that have been used in prior studies to provide valid estimates of two key components of social intelligence: 1) Sensitivity to others and 2) confidence in one’s capacity to manage social situations. Pre- and Posttest were administered to participants in the SI training, and also to 87 students in similar classes who did not receive the program who served as the control group. Gender and emotional intelligence levels at pretest also were examined as potential individual differences that might affect the impact of the program on study outcomes. Repeated measures ANOVAs on study outcomes revealed significant increases, from pre to post, in most measures of social intelligence for program participants in comparison to controls, with no effects of gender or age on program effectiveness. Prior scores on emotional intelligence were not a prerequisite for learning from the program. Some findings suggest ways the program may be improved to have stronger effects. Nonetheless, the findings indicate that the SI program tested here shows considerable promise as a means to increase the willingness of young adults to take the perspective of others and enhance their efficacy for initiating and sustaining positive social connections.

## Introduction

Can people be taught to attend to one another with greater care? Can a person’s social relations improve with training? Specifically, will students who participate in a social intelligence training program show a greater sensitivity to the emotions of others [1, 2], an enhanced willingness to take the perspective of others, [3] and show more confidence in their ability to successfully engage in social relations [1]? If so, does this capacity to learn vary as a function of gender or prior emotional skills? These questions are the focus of this study.

### The value of healthy relationships with others

We all agree that people should treat one another with humanity. Aside from the everyday moral purpose served, being “good” to others is good for us. Empirical evidence from many sources confirms a strong association between social functioning and the physical health of people of all ages. Individuals with strong social relationships have healthier cardiovascular functioning, more efficient immune responses to pathogens, they suffer less disability in response to illness, and they live longer [4–8]. Further, emotional health depends on quality social relations. Anxiety and depression are linked to lost or threated social bonds, and those without social connections are at greater risk of suicide [9]. Finally, being able to successfully get along with others is necessary for our survival. As Humphrey [10] has observed, we are social animals. Without the ability to perceive the intentions of other animals, and influence their actions, we would not survive.

### Disengagement and disregard of others is prevalent

In spite of the established value of social connection, there is mounting evidence that in Western industrialized nations, young adults have difficulties in relating to others [11]. There also are signs of increased disregard for one another among young adults. In a meta-analysis of 72 empirical studies conducted over the past 30 years, Konrath, O'Brien, and Hsing [12] reported a 34% drop in perspective-taking in college students, with the steepest declines over the last 10 years. In addition, abusive social interactions appear widespread. In their studies of bullying at school, Wang, Iannotti, & Nansel [13] found that as many as 1 of 7 adolescents are repeatedly targeted by their peers for mistreatment. While the majority of these studies have been conducted in the United States, researchers from European nations have also called for more attention to the prevalent problems of mistreatment of one another among their nation’s youth [14].

### Can the high rates of disengagement and disregard be reduced?

Though the benefits of social connection are well-established, and the prevalence of social disconnection high, little is known about the mutability of the capacity to be social in later adolescence and early adulthood. These styles of relating may be fixed early in life, and unresponsive to efforts to enhance social facility, except perhaps with intensive psychotherapy [15].Evidence of the effectiveness of social-emotional learning programs has been reported for programs for children [16], though some reviews cast some doubt about the claims of success [17]. Few SEL programs for teenagers and young adults have been developed and tested. To our knowledge, no evaluation of a program that focuses on the development of social relations for this age group has yet appeared in the literature. In this paper we report on the first empirical test of a public health intervention designed to reverse the trends of disregard and disengagement among young adults through educational training in social intelligence.

### What is Social Intelligence (SI)?

First introduced by Thorndike [18], SI refers to a keen awareness of the value of social connections, the ability to take another’s perspective, and the capacity to engage in satisfying relationships [19]. Whereas most definitions of SI are close to, if not fully synonymous with, social competence, [20–21], there also is an important person-centered element, a commitment to reducing the objectification of others and fostering “humanization” of relationships [22]. Though the term “intelligence” connotes a stable ability, most investigators define SI not as a fixed trait, like IQ, but an organized set of cognitive principals that permit accurate judgments and wise choices in one’s interactions–a capacity that develops with thoughtful reflection about oneself, combined with a greater understanding of one’s social world, as well as an enhanced motivation to become less self-focused and more attentive to others [23, 20, 24].

Emotional intelligence (EQ) is a construct that is often used in conjunction with SI. Salovey & Mayer [25] define EQ as the ability to monitor one’s own and others’ feelings, to discriminate among them, and to use this information to guide one’s behavior. As the listing of the component abilities indicates, emotion regulation is frequently evoked in a social context, and EQ programs have been shown to successfully engage people in the practice of greater awareness of not only one’s own feelings and motivations, but also the feelings and motivations of others [26, 27]. However, the focus of EQ programs is still narrowly on the “self”. They do not target the person’s approach to their social worlds directly, which may be what matters most in sustaining good health and sense of well-being. A recent test of an emotional intelligence program conducted in Spain for adolescents provides one illustration. While decreases in reports of negative emotions such as hostility were found, the evidence of changes in perspective-taking, as well as other indices of empathy, were not uniform, showing improvement primarily with males [14].

In contrast to emotional intelligence programs, a modern SI program draws from social neuroscience findings to assert that we are, by definition, relational beings [28]. As Lieberman [29] has pointed out, most exciting life experiences are social and most intrinsically meaningful activities involve people closest to us. Indeed, the most distressing life events involve other people, whether it is disconnection due to loss, disaffection through divorce, or the threatened loss of humanity through witnessing or being a victim of abuse. SI interventions can inform people of how central their interpersonal world is in their lives, encouraging them to broaden their capacity to form and sustain social connections, and repair damage done by past experience in troubled relationships.

The social intelligence training we examine in this study was designed to modify key social cognitions regarding social engagement, enhance efficacy expectations regarding performance in interpersonal relations [30], and encourage the adoption of core values associated with the humanization of social relations [21]. In addition, the approach includes attention to potential barriers to social-emotional development due to adverse childhood experiences [22, 31] and insecure attachment, which is prevalent in Western cultures [32].

### Study Questions

This investigation tested whether participants receiving the SI curriculum would improve on measures of social intelligence. We hypothesized that students would report greater awareness and sensitivity to others, increased willingness to take the perspective of others in their daily lives, and enhanced self-efficacy.

Although we did not predict we would find effects, we examined evidence for individual differences in program effects due to EQ and gender. Is sufficient capacity to understand one’s own emotions and the emotions of others a prerequisite to the development of social capacities [33]? To probe this question, we investigated whether those with low initial scores on a measure of EQ would respond differently to the SI program. We also wondered whether cultural influences on social identity based on gender roles would influence receptivity to the SI curriculum. Thus, gender was examined as a personal characteristic that might distinguish those who improve in SI from those who do not gain from the program.

## Methods

### Participants

The three-month SI program was administered at the Universidad Rey Juan Carlos in Madrid, Spain, with complete outcome data available for 207 students who took part in the applied psychology section of a course in “General Psychology” for medical and physiotherapy students, or during a “Learning Psychology” sequence for teacher training program students in the Fall semester of 2013. The control group with complete outcome data was comprised of 87 students from the same university (Rey Juan Carlos) and the same mix of majors: Medicine, Physical Therapy, and Teacher Education drawn from classes held in the winter and spring of 2014. In addition to the participants with complete data, 19.3% (n = 40) of those originally assigned to the experimental group had missing pre or post scores as well as and 16.1% of controls (n = 14). Table 1 displays the gender and age distributions, mean age, and total scores at pretest on emotional intelligence across the groups, including those who were dropped from the final analysis. Chi-Square-tests of differences between groups on age (% greater than 24 years of age) and gender were non-significant (p’s > .10). One-way ANOVA’s revealed no significant differences between participants with and without missing data on pre scores on emotional intelligence. However, as shown i n Table 1, there was a difference in mean age, across the four groups. Post hoc Scheffe contrasts revealed one significant difference: Experimental participants without missing data were younger than those assigned but dropped from the experimental group due to missing (p < .05). 2.75). This difference between groups in mean age prompted examination of age as a possible influence on study findings through covariance analyses noted below.

**Table 1:** Gender, Age and Emotional Intelligence (EQ) Score Distributions across groups.

	Experimentals (E) (N = 207)	Controls (C) (N = 87)	Dropped E’s (N = 40)	Dropped C’s (N = 14)	x^2^/ F (df)
Gender % Female	82.0%^a^	73.3%^a^	85.0%^a^	100%^a^	6.75 (3)
Age % < 25 years	90.8%^a^	91.9%^a^	87.5%^a^	92.9% ^a^	.70 (3)
Mean age (sd)	20.65^a^ (4.5)	21.42^a^ (2.8)	23.08^b^ (7.6)	19.71^a^ (2.6)	3.76* (3, 342)
EQ (sd)	3.09^a^ (.58)	3.18^a^ (.56)	3.11^a^ (.69)	3.08^a^ (.78)	.56 (3, 318)

Notes: x^2^ (3): F (3,314–342);

*p < .05

Values with the same superscript ^a^ are not significantly different from each other

11 Participants did not report gender; 2 participants did not report age.

### Procedures following enrollment

The students in the experimental condition were instructed to view the online program in their homes, and were provided the website and a password to gain access. The students in each class were given two weeks to view one of seven modules and prepare written reflections in response to awareness exercises that followed each session in the module. Every two weeks, the students discussed their reflections with one of the study authors in their classroom and participated in exercises that reinforced the content of the modules. All participants completed a set of questionnaires both before and after they completed the program. Control participants filled out these measures at the beginning of their classes and three months later to coincide with the time-span between assessments for participants in the experimental condition.

### Ethics Statement

The authors assert that all procedures contributing to this work comply with the ethical standards of the relevant national and institutional committees on human experimentation and with the Helsinki declaration of 1975, as revised in 2008. The study protocol was first approved on 2/13/2013 by the Institutional Review Board of the Office of Research Integrity & Assurance of Arizona State University, Tempe, Arizona, USA (Protocol # 1302008821). Written informed consent was obtained from all participating students at Rey Juan Carlos University in Madrid, Spain by the third and fourth authors, who then provided de-identified data to the first author for analysis in the United States. All participants were adults, 18 years of age or older; thus no additional parental consent was sought.

### Social Intelligence Intervention

#### Core social cognitive principles

Four meta-cognitive principles provided the foundation for the curriculum. The first principal, Humanization, was the value orientation that guided the training throughout [23, 21]. Underlying this principle is the importance of treating one another not as objects to manipulate, but as people with cares and concerns worthy of our attention.

The second principle that guided the program elements was that attention to lessons learned from cognitive and social neuroscience are necessary to inform our awareness of, and behavior toward, one another. Here, the program relied on evidence from cognitive science that the mind simplifies, classifies, and automatizes our thoughts and our behaviors toward ourselves and others. Our interactions with others, guided by these schemas (concepts which inform a person about what to expect from situations) and heuristics (experience-based techniques for problem solving, learning, and discovery used to find a “good enough” solution) [34], are beneficial, but also potentially harmful to our aims to improve relationship quality and treat one another with humanity. Each person has the opportunity to replace less optimal, relational schemas and heuristics with those that enhance social capacities [20], within limits imposed by cognitive capacities. The brain’s ability to form new connections, often referred to as neuroplasticity, underscores the key lesson that people can modify habitual thoughts and actions to resolve their difficulties in social relationships and further their interests in improving their social relationships.

The third principle that guided the curriculum was that each person is unique, shaped by past experiences and expectations for the future. This principle introduced a natural tension between the humanization principal and the mind’s tendency to simplify. Since no two people are alike, it is the case that our understanding of them is incomplete, short-sighted, and biased to some degree. Such biases are amplified by in-group identification and denigration of out-group members [35, 13]. SI training was designed to raise awareness of these challenges and offer a way to overcome biases toward others, and rigid adherence in social schemas that are established early in life.

The fourth principal that informed the SI curriculum was that of choice. People naturally seek connection with others, and to do so, need to understand one another [36–38, 28], and develop a theory of mind that permits taking another’s perspective [39–40]. However, it is not enough to intellectually understand these concepts. One must put them into practice and that is a choice we need to make everyday

### Program Elements

The four meta-cognitive principles that guided development of the online curriculum were organized around seven thematic modules. Module 1 introduced the program, defined humans as fundamentally social creatures, described the importance of SI training, and presented the basic principles that guide the training. Module 2 described how the social brain processes information about ourselves and others, guided by schemas and simple rules of thumb called heuristics, and how greater awareness of the utility and fallibility of these ways of thinking can improve our capacities to understand ourselves and others [34]. Module 3 focused on empathy–the ability to identify feelings and thoughts of another person and respond to those feelings and thoughts appropriately. Also addressed in this session are the teachable capacities to fully grasp the perspectives of others, predict how others will act, and how others will feel about our actions [41–43]. Important limitations to compassionate responding were introduced in Module 4, which addressed how our thoughts and behavior toward others are shaped by in-group, out-group biases, many outside of awareness [44–46]. These sessions were designed to raise awareness of the nature of prejudice, and introduce ways of responding to out-group members more intelligently [47–48]. Module 5 presented the contrast between the plentiful but relatively shallow connections formed online compared with the potential richness of in-person connections [49–50]. Module 6 discussed the ebb and flow of positive face-to-face social interactions, factors that disturb that natural cadence, and the fundamental importance of getting outside of our own heads and connecting with others [24, 51]. Module 7 addressed both how our backgrounds and past experiences, particularly interactions with parents and other family members early in life, shape schemas formed about the trustworthiness of social relationships, and our willingness to engage [15, 52]. However, the module also reminded the student that people also have the capacity to form new social connections [53–56], modify our schemas once we are aware of them, and enhance the quality of long-standing relations in need of repair.

### Delivery method of Intervention

Considerable advances in web-based instructional methods have been made, paralleling technological advances permitting widespread access to high capacity internet [57]. Recent reviews identified communication training programs as among the most successful online behavior change interventions [58]. Web-based interventions have shown comparable efficacy [59] and even outperformed face-to-face interventions in some cases [60]. Web-based programs vary significantly in attrition rates [61–62] and degree of influence over behavior.

Program materials were shaped to both sustain attention and enhance learning. Construction of the SI audio/visual training program followed three "deep learning" (rather than "shallow learning") concepts. First, the SI course was developed based on the fact that information cements into long-term memory by 1) gradually presenting new material, then repeating it throughout the course, 2) lacing content with real world examples to provide meaning to learners, and 3) engaging learners' multiple senses using colorful images, narration, and animation, rather than text alone. [63] The second deep learning concept is that learners must stay engaged with the material without information overload. Prior research has shown that the average engagement time of any internet video maxes out at 6 minutes, regardless of the length of the video [64]. SI videos averaged 5.5 minutes in length. Finally, learners are guided away from passive reaction to their world by actively testing the SI principles through observation, experimentation, and conclusions about themselves and others. At the end of each session, participants were prompted to write their responses to awareness-raising questions designed to provoke thoughtful attention to relevant current and past personal experiences.

### Study Measures

EQ was assessed with the Spanish version [65] of the Trait Meta-Mood Scale [25], a 24-item self-report instrument using a five-point Likert scale (from 1 to 5). Scale items are shown in Table 2. The scale is comprised of three sub-scales to assess three central components of EQ: the extent to which the person attends to his/her feelings, the degree to which the person clearly perceives his/her own emotional states, and the person’s capacity to use cognitive strategies to repair negative mood. The TMMS measure of EQ has shown high internal consistency and satisfactory test–retest reliability in past studies [25], and showed good internal consistency reliability in the current study (alpha = .90). A median split on pre-scores on EQ was used to identify groups with low versus high scores on this measure.

**Table 2:** Emotional Intelligence (EQ).

I don’t pay much attention to my feelings.
I can never tell how I feel.
I usually know my feelings about a matter.
Sometimes I can’t tell what my feelings are.
I can’t make sense out of my feelings.
I don’t have much energy when I am happy.
I think about my mood constantly.
I often think about my feelings.
I pay a lot of attention to how I feel.
I am usually very clear about my feelings.
I am rarely confused about how I am feeling.
I never worry about being in too good a mood.
I am often aware of my feelings on a matter.
I almost always know exactly how I am feeling.
I don’t let my feelings interfere with what I am thinking.
I don’t usually care much about what I’m feeling.
It is usually a waste of time to think about your emotions.
When I’m angry, I usually let myself feel that way.
No matter how badly I feel, I try to think about pleasant things.
When I become upset, I remind myself of all the pleasures in life.
I try to think good thoughts no matter how badly I feel.
If I find myself getting mad, I try to calm myself down.
Although I am sometimes sad, I have a mostly optimistic outlook.
I don’t think it’s worth paying attention to your emotions or moods.

English language version of theTMMS-24 items used to assess emotional intelligence. Scale adapted from**: [25]**

Two aspects of social intelligence were assessed pre and post intervention: 1) Sensitivity to others and 2) Confidence in one’s own capacity to manage social situations. These two primary aspects of social intelligence were assessed with four self-report instruments: The Tromsø Social Intelligence (SI) Scale [1], the revised 13-item Snyder Self-Monitoring Scale (SSM) [2], the perspective-taking sub-scale from the Davis Interpersonal Reactivity Index (IRI) [3], and the General Self-Efficacy Scale [66]. Table 3 provides a listing of the scales and accompanying items that constitute measures of Sensitivity to others. Table 4 shows the measures and accompanying items that provide assessments of Confidence in Social Situations.

**Table 3:** Sensitivity to Others.

**SSM- Social Sensitivity**
In conversations, I am sensitive to even the slightest change in the facial expressions of the person I’m conversing with.
My powers of intuition are quite good when it comes to understanding other’s emotions and motives.
I am often able to read people’s true emotions correctly through their eyes.
I can usually tell when I’ve said something inappropriate by reading it in the listener’s eyes.
If someone is lying to me, I usually know it at once from that person’s manner of expression
I can usually tell when others consider a joke to be in bad taste, even though they may laugh convincingly.
**IRI: Perspective-taking**
I sometimes find it difficult to see things from the “other guy’s” point of view.
I try to look at everybody’s side of a disagreement before I make a decision.
I sometimes try to understand my fiends better by imagining how things look from their perspective.
If I’m sure I’m right about something, I don’t waste much time listening to other people’s arguments.
I believe that there are two sides to every question and try to look at them both.
When I’m upset at someone, I usually try to “put myself in his shoes” for a while.
Before criticizing somebody, I try to imagine how I would feel if I were in their place.
**SI-Social Information Processing**
I can predict other peoples’ behavior.
I know how my actions will make others feel.
I understand other people’s feelings.
I understand other’s wishes.
I can often understand what others are trying to accomplish without the need for them to say anything.
I can predict how others will react to my behavior.
I can often understand what others really mean through their expression, body language, etc.
**SI-Social Awareness**
I have often hurt others without realizing it.
I find people are unpredictable.
I often feel that it is difficult to understand others’ choices.
It seems as though people are often angry or irritated with me when I say what I think.
People often surprise me with the things they do.
Other people become angry with me without me being able to explain why.
I am often surprised by others’ reactions to what I do.

English language version of the items of the four subscales used to assess sensitivity to others. Scales adapted from: [2]. [3]. [1]

**Table 4:** Self-Confidence in the Capacity to Manage Social Situations.

**SSM-Self-regulation**
I have trouble changing my behavior to suit different people and different situations.
Once I know what the situation calls for, it’s easy for me to regulate my actions accordingly
In social situations, I have the ability to alter my behavior if I feel that something else is called for.
I have found that I can adjust my behavior to meet the requirements of any situation I find myself in.
Even when it might be to my advantage, I have difficulty putting up a good front.
I have the ability to control the way I come across to people, depending on the impression I wish to give them.
When I feel that the image I am portraying isn't working, I can readily change it to something that does.
**General Self-Efficacy Scale (GSES)**
I can always manage to solve difficult problems if I try hard enough.
If someone opposes me, I can find means and ways to get what I want.
It is easy for me to stick to my aims and accomplish my goals.
I am confident that I could deal efficiently with unexpected events.
Thanks to my resourcefulness, I know how to handle unforeseen situations.
I can solve most problems if I invest the necessary effort.
I can remain calm when facing difficulties because I can rely on my coping abilities.
When I am confronted with a problem, I can usually find several solutions.
If I am in a bind, I can usually think of something to do.
No matter what comes my way, I’m usually able to handle it.
**SI-Social Skills**
I often feel uncertain around new people who I don’t know.
I fit in easily in social situations and meeting people for the first time.
I am good at entering new situations and meeting people for the first time.
I have a hard time getting along with other people.
It takes a long time for me to get to know others well.
I am good at getting on good terms with new people.
I frequently have problems finding good conversation topics.

English language version of the items shown here. Scales adapted from: [2]. [1]. [66]

The Tromsø SI Scale consists of 21-items yielding a total score and three 7-item subscale scores of social intelligence: SI-Social information processing (alpha = .75), designed to assess the ability to understand and predict others behaviors and feelings, SI-social skills (alpha = .79), which assesses the ability to enter new social situations and adapt to them, and SI-social awareness (alpha = .70), assessing the degree of awareness, and lack of surprise in response to social situations. Students rated on a 5-point scale how well they were able to perform each skill (1 = not at all like me, 5 = very much like me). Alpha reliability for the total score was .80 for this sample.

The SSM [2] was comprised of two sub-scales: SSM- sensitivity to expressive behavior of others (six item; alpha = .68) and a seven-item sub-scale, SSM-self-regulation (alpha = .74), that measures he ability to modify one’s own behavior when socially appropriate. For both subscales, students provided ratings on a 5-point scale: 1 = not at all like me, 5 = very much like me. The seven-item sub-scale from Davis’s IRI [3] assessed perspective-taking (alpha = .72), and the Spanish version of the ten-item General Self-Efficacy Scale (alpha = .86) [66, 67] was used to assess expectations for success resolving difficulties. Pre and post scores on each of these scales constituted study outcomes. In addition, two composite scores were calculated by summing item mean scores for the scales assessing Social Sensitivity and Social Self-Confidence (see Tables 3 & 4 for scales designated for each composite).

### Approach to Data Analysis

The approach to the analysis of differences in outcomes between groups was conducted in stages. First a series of t-tests of differences between groups on scores at pretest were conducted to assess degree of equivalence between the experimental and control groups. Pre and post scores of the four measures of social intelligence were then examined through separate repeated measures ANOVAs with one within-subject factor (time), and two between-subject factors: Treatment condition (intervention or control) and EQ (high or low) at pretest. (Gender and age also examined initially for potential effects alone, and in interaction with treatment condition and EQ to predict pre-post changes on study outcomes. No significant effects were found on treatment outcomes in this college sample due to gender or age so they were removed in the final ANOVA tests of group differences over time. These ANOVAs were supplemented by ANCOVA’s for scales with pretest differences between conditions, and regression analyses which allowed the full range of scores on EQ to be used to test for main effects and interaction effects of EQ and condition to predict outcomes. No substantial differences in the results were found when using this alternative analysis framework, so only the repeated measures ANOVA results are presented here.

## Results

### Group Equivalence on outcome measures

A series of t-tests across all outcome measures was conducted to test for differences between experimental and control groups at pretest. No significant differences between groups at pretest were found for the total scores on Emotional Intelligence and the Tromsø SI scale, SI-social skills, the IRI-perspective-taking, and the SSM-self-regulation scales. Controls reported higher scores at pretest on General Self-Efficacy (p < .05), the SSM-social sensitivity (p < .01), and the SI-Social Information processing sub-scale (p < .01). Controls reported lower scores on SI-social awareness (p < .01).

### Social Intelligence

Table 5 summarizes the results of the pre-post differences uncovered in the repeated-measures data analyses for the two primary aspects of social intelligence: Sensitivity to others, and Confidence in one’s own capacity to manage social situations. As the table shows, significant increases in social intelligence pre to post were observed across groups favoring the Experimental condition on measures gauging sensitivity to others, with the exception of the SI- SI-social awareness subscale and on measures assessing social self-confidence. Effects for the Condition by Time interaction were small in size, but did show some variability across scales.

**Table 5:** Repeated Measures ANOVA Results for all outcome measures.

	Experimentals		Controls		ANOVA Results			
	Pre $\bar{\text{x}}$ (sd)	Post $\bar{\text{x}}$ (sd)	Pre $\bar{\text{x}}$ (sd)	Post $\bar{\text{x}}$ (sd)	Time (T)	Group (G)		G x T	Partial η^2^For G x T
**A. Sensitivity to Others**								
SSM-Social Sensitivity	3.31 (.63)	3.60 (.78)	3.67 (.54)	3.63 (.78)	9.33**	6.01*	14.97***	.049
IRI- Perspective Taking	3.66 (.61)	3.82 (.62)	3.74 (.54)	3.76 (.55)	12.03***	.00	6.74**	.023
SI-Social Info. Processing	3.42 (.54)	3.60 (.54)	3.70 (.36)	3.67 (.39)	8.22**	.002	14.10***	.047
SI- Social Awareness	3.62 (.60)	3.69 (.60)	3.34 (.45)	3.38 (.50)	3.37	20.64***	.215	.000
Sensitivity to others composite score	3.50 (.41)	3.68(.45)	3.62 (.32)	3.61 (.36)	18.87***	.08	21.51***	.069
**B. Social Self-Confidence**								
Self-Efficacy	3.01 (.44)	3.15 (.43)	3.12 (.34)	3.08 (.35)	7.04**	.09	22.38***	.071
SSM- Self-Regulation	3.40 (.63)	3.60 (.66)	3.36 (.41)	3.39 (.45)	18.65***	3.87	8.62**	.029
SI- Social Skills	3.51 (.73)	3.61 (.68)	3.59 (.40)	3.56 (.47)	1.45	.00	5.38*	.018
Social Self-Confidence composite	3.31 (.48)	3.45 (.48)	3.36 (.27)	3.35 (.30)	13.99***	.47	17.96***	.058
**C. Emotional Intelligence (EQ)**								
EQ	3.09 (.58)	3.19 (.62)	3.18 (.56)	3.20 (.55)	5.78*	.52	3.01 ^a^	.01

Notes: F’s (1, 288–296); df’s vary due to missing values on some indicators

^a^ p < .10

*p < .05

**p < .01

*** p< .001

**Fig 1:**
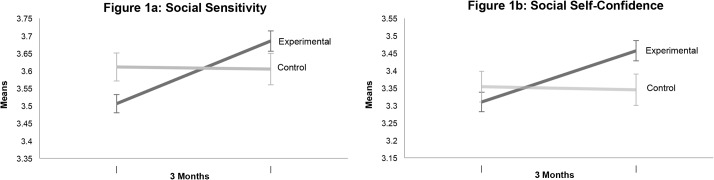
Pre and Post Means with Standard Error Bars for Experimentals and Controls on Social Sensitivity and Social Self-Confidence Composites.

Table 5 also provides results from the repeated measures ANOVA’s for the two composite indices for Social Sensitivity and Social Self-Confidence. Group by Time interactions shown there revealed small but highly significant increases in social intelligence pre to post for the experimental group in comparison to controls [Social Sensitivity; partial eta^2^ = .069; Social Self-Confidence; partial eta^2^ = .58] . These findings are graphically displayed in Fig 1.

### Post-hoc Tests

The repeated measures analyses of variance do not fully examine how the results differed between experimental and control groups when examining effects over time. A series of paired t-tests were run on all outcomes reported in Table 5, separately for experimental participants and controls. All t-tests revealed significant increases on outcomes (p’s < .01) pre to post for experimental participants except the SI-social awareness sub-scale where the effect was marginal (p < .10). For the controls, none of the paired t-tests revealed significant increases on outcomes, pre to post, and one measure, generalized self-efficacy, showed a significant decline (p <.01). These findings should not be interpreted to mean the posttest scores were always significantly higher for the experimental groups, as these gains were relative to their pre-scores. For example, post-test only comparisons of scores on the composites revealed significant differences for Social Self-confidence (p < .05), but only marginal differences between groups for Social Sensitivity (p < .10, one-tailed test). When applying appropriate controls for pre-test levels with ANCOVA’s, though, results replicated findings in the repeated measures ANOVA’s: significantly greater gains on both composites for the experimental group compared to controls.

### Emotional Intelligence and Social Intelligence

Emotional Intelligence scores at pretest did not moderate program effects. (All EQ by Time and EQ by Time by Condition effects were non-significant: p’s > .10). The repeated measures ANOVAs test of between group differences, however, did reveal that participants who rated their social intelligence highly, also rated their EQ highly, both pre and post the intervention on most scales. Some of these relationships were particularly strong. F’s (1, 288) ranged from a low of 1.92 on the SI-social awareness sub-scale to an F (1, 288) = 44.05 on SI-social information processing. The total score from the Tromsø SI measure across pre-post measurement, for example, showed a highly significant between-group difference favoring those high on EQ (F = 23.05, p < .001; partial eta ^2^ = .07).

To examine whether the SI training had any effects on EQ scores, we administered the Trait Meta-Mood Scale of EQ at posttest as well as at pretest, and performed a two-way repeated measures ANOVA. The results of this analysis are shown in Table 5. A main effect for time was observed (p < .05) suggesting that both groups appeared to increase in reports of EQ over time, with only a marginally significant increase in total EQ for that was greater for the intervention group, as revealed in the Condition by Time interaction (p <.10). Closer inspection indicated a slight but statistically significant change on one subscale of Emotional Intelligence: Greater attention to emotions pre to post for the experimental group in comparison to controls, F(1,292) = 5.44, p < .05), but no change on the other two sub-scales of EQ (p’s > .15).

## Discussion

Our results addressed three key research questions: 1. Does an SI training course improved a) social sensitivity, and b) social self-confidence: 2. If people initially low in EQ respond differently to training compared to people initially high in EQ?: 3. Whether males and females respond differently to training., and 4. If the SI program also had effects on EQ The findings provide consistent evidence that the SI curriculum led students to enhance their sensitivity to other people’s, and develop greater confidence in their ability to successfully navigate social relations. Higher scores pre to post following administration of the SI program compared to controls were observed on all but one of the outcomes assessing social facilities. The findings did not vary as a function of prior levels of emotional intelligence, and were not more or less effective for men compared to women. The findings testing for effects on EQ were equivocal; suggesting the program tested had, at best, only a marginal effect on how a person attends to their own emotions.

There was variability in the sizes of the effects across measures. Though all significant effects were small, the partial eta-squared for the Tromsø SI- social information processing was more than twice that of the Tromsø SI-social skills sub-scale, and there were no statistically significant changes pre to post on SI-social awareness. The lack of findings on this subscale may be due to the program module on unconscious determinants of social behavior. There, participants are taught that many of their thoughts and actions in social situations occur outside of their awareness. This information runs counter to the content of items on the awareness measure, which assesses the degree to which the respondent *is* aware of the effects of how his/her behaviors and others’ behavior. (See Table 2 for a listing of the items on the SI-social awareness sub-scale.) It appears that revisions in program content may be warranted to ensure that participants understand that they are capable of increasing their awareness of how they affect others, and that it is beneficial to do so. The program discusses how most troublesome social behavior is often unconscious, and not intended to be harmful; a lesson that is designed to reduce unnecessarily harsh social judgments. Preservation of that theme would be important when undergoing program revisions designed to teach students how often we are unaware of our actions and reactions in social situations. There also may be other modifications to the current program worth considering that would increase program impacts. The effects on social skills were modest, and more engaging program exercises appear necessary to improve program impacts. The ordering of the program elements also is worth considering. In light of recent dramatic demonstrations of the importance of teaching how the brain changes with new learning [68], greater attention to neuroplasticity concepts early in the program may strengthen the intervention’s effects. We urge further study on this and related issues.

### Emotional Intelligence and SI

The data do not indicate that emotional understanding is necessary for people to learn to better manage their social worlds. EQ and SI are closely-related constructs, and prior studies of online interventions have shown that raising emotional awareness can improve social relations [27]. Future social intelligence interventions including revisions of the program tested here would likely have stronger effects on both social and emotional concerns if more explicit attention were given to emotional regulation within the context of social development. What is apparent from this study is that the two types of intelligences, sometimes conjoined as social-emotional facilities, are separate constructs, and that an intervention tailored to influence one does not automatically influence the other. Programs that target social relations may do so successfully without influencing emotional capacities, and emotion regulation programs also cannot be assumed to take care of problems in social regulation.

### Other Factors

Individual differences other than gender and EQ may affect the impact of the SI training evaluated in this study. For example, the age range in the current study was narrow, precluding careful testing of program effects that may vary across the life-span. Furthermore, those with troubled childhoods often have a more difficult time forming and retaining positive social ties throughout life and have difficulty with emotion regulation [69–71]. Indeed, the capacity to navigate through complex social relationships, and a willingness to engage, is built from a foundation of close family relationships early in life. Engrained schemas of distrust and insecure attachment [15], formed from parental neglect and/or abusive relations in childhood, could diminish readiness to learn from the SI program. However, the absence of a secure attachment early does not preordain a lifetime of difficulties with relationships [72–74], and may even heighten sensitivity to positive interpersonal experiences such as those introduced in the program [75]. Future work needs to address the extent to which people of diverse backgrounds can be reached through public health education programs like this one, and the degree to which those changes have an influence on psychological well-being and physical health.

## Limitations

This initial evaluation of the SI program effectiveness leaves several questions unanswered about the nature of the effects observed. First, the study design did not include a randomized control condition, so pre-existing differences between groups may have influenced the findings. The control group data were collected on a subsequent semester, and it is conceivable that differences in social maturity could play a part in the findings. Would the experimental participants look like the controls eventually even without the intervention? We think not. The two groups were equivalent in age and gender composition, and did not show pretest differences on many of the study measures for which we did find differences pre to post. Further, there was no evidence of ceiling effects on control group scores; the scales used to assess outcomes showed sufficient variability at pre and posttest for both groups. Nevertheless, there were differences between groups, on some scales, three showing pretest differences that were higher for the control group, and one pretest difference favoring the experimental group. Supplementary analyses of the data including ANCOVA tests do add to our confidence that the gains in social intelligence were due to the receipt of the intervention, and not from other differences between groups, but the lack of ransom assignment remains a limiting factor in judging the significance of the findings.

Posttest sensitization due to expectations of benefit cannot be ruled out as possible limitation on the generalizability of study findings. The self-report methods are particularly susceptible to such influences. It is interesting to note that the Tromsø social-awareness sub-scale, a variable that might have been sensitive to expectancy and/or testing effects, did not change from pre- to posttest. This finding, though a concern to address in future revisions of the program, does lend support to the interpretation that changes observed were specific to the program, and not due to some generalized expectancies regarding the program’s value. Nevertheless, future studies are needed for which random assignment to condition is in place, and observer ratings in addition to self-reports are included when evaluating program effects. Longitudinal studies also are needed to estimate the extent to which the improvements in SI found here would yield lasting benefits. The SI sessions were each designed to be brief but “wise” interventions, built to set in motion changes in perspective about oneself and others over time, resulting in long-term changes in social behavior [[76]. Future tests of the program could include follow-up assessments by peers on social intelligence of the participants who received the program.to examine whether such effects emerged over time.

It is important to note that the generalizability of study findings is limited. Only Spanish college students were studied here; a young, well-educated, European sample who were in classes for which the SI curriculum was part of their course requirement. Future studies are needed to test the range of the applicability of these findings to other demographic groups.

## Conclusions

In sum, the findings indicate that this program in SI shows considerable promise as a means to increase the capacity of young adults to develop and sustain positive relationships. Students who engaged in this program, built from evidence-based research in social neuroscience, cognitive psychology, and social psychology, increased their attention to others’ emotions, exhibited a greater willingness to take the perspective of another, and reported a greater self-efficacy in social situations than controls. Though program effects were modest, they were consistent across study outcomes, and did not depend on pre-existing levels of emotional intelligence. Though this initial study has limitations that urge caution in the interpretation of the findings, the results clearly support further examination of how this SI curriculum, and others like it, can advance the social well-being of people in western cultures.

## Supporting Information

S1 File Study Data. (SAV)

S2 File Data Code Book. (DOCX)
