# Supplementary material for: Can We Learn to Treat One Another Better? A Test of a Social Intelligence Curriculum
Source: PLoS One. 2015 Jun 15;10(6):e0128638. doi: 10.1371/journal.pone.0128638 (PMC4468120; doi:10.1371/journal.pone.0128638)
Supplement: S2 File — (DOCX) [file pone.0128638.s002.docx]

Supplementary File 2: Data Code Book

**NOTE: This codebook is for PLOS one to accompany re-submission 4-11-2015**

**Demographics**

| **Variable** | **Item in English** | **Item in Spanish** | **Scale** |
| --- | --- | --- | --- |
| Nsujeto | Subject number |  |  |
|  |  |  |  |
| BBDD |  |  | 1=Experimental  2=Control |
| fecha | Date | fecha | Day.month.year ? (Open field?) |
| Nombrey apell | Name | NOMBRE Y APELLIDOS | Student’s name (Open field) |
| DNI | ? (Possibly a student ID, was it random) |  |  |
| Sexo | Gender | Sexo | 1=Female  2=Male |
|  |  |  |  |
|  |  |  |  |
|  |  |  |  |

Revised **Snyder’s Self-Monitoring Scale (SM): Subscales:** Ability to modify self-presentation (M) and Sensitivity to expressive behavior of others (S)

Lennox & Wolfe (1984). Revision of the Self-Monitoring Scale. Journal of Personality and Social Psychology, 46, 1349-1364.

[Measures spain\Self-monitoring\revision_of_the_self-monitoring_scale..pdf](file:///C:\Users\atajz\Documents\Dropbox\SI%20Carmen,%20Alex%20and%20Eva\Measures%20spain\Self-monitoring\revision_of_the_self-monitoring_scale..pdf)

[Measures spain\Self-monitoring\self-monitoring scales lennox & wolfe 1984.pdf](file:///C:\Users\atajz\Documents\Dropbox\SI%20Carmen,%20Alex%20and%20Eva\Measures%20spain\Self-monitoring\self-monitoring%20scales%20lennox%20&%20wolfe%201984.pdf)

Scale in English Scale in Spanish

1. Not like me at all! 1 NO! En absoluto es como yo

2 2

3. Somewhat like me 3 Algo parecido a mi

4. 4

5. Very much like me 5 SI! Muy parecido a mi

| sm=SELFMONITOR_SI (Ability to modify self-presentation)  so=SENSITEXPRESSBEH_SI (Sensitivity to expressive behavior of others)   \| Variable \| Subscale \| Survey \| Item \| Spanish \| \| --- \| --- \| --- \| --- \| --- \| \| CIS2_so \| so \| 2 \| In conversations, I am sensitive to even the slightest change in the facial expressions of the person I’m conversing with \| En una conversación soy sensible incluso al mínimo cambio en la expresión de la cara de la persona con la que estoy conversando. \| \| CIS3_so \| so \| 3 \| My powers of intuition are quite good when it comes to understanding other’s emotions and motives. \| Mis poderes de intuición son muy buenos a la hora de entender las emociones y las motivaciones de los demás. \| \| CIS5_so \| so \| 5 \| I am often able to read people’s true emotions correctly through their eyes \| A menudo soy capaz de leer las verdaderas emociones de las personas a través de sus ojos. \| \| CIS9_so \| so \| 9 \| I can usually tell when I’ve said something inappropriate by reading it in the listener’s eyes \| Puedo saber cuando he dicho algo inapropiado leyendo en los ojos de la otra persona. \| \| CIS10_sm  Reversed \| sm \| 10 \| I have trouble changing my behavior to suit different people and different situations \| Tengo problemas para cambiar mi comportamiento y adaptarme a diferentes personas en diferentes situaciones. \| \| CIS13 \| sm \| 13 \| Once I know what the situation calls for, it’s easy for me to regulate my actions accordingly \| Una vez que sé lo que la situación requiere, es fácil para mi regular mis acciones en consecuencia. \| \| CIS16 \| so \| 16 \| If someone is lying to me, I usually know it at once from that person’s manner of expression \| Si alguien me esta mintiendo, normalmente lo se desde el principio por su manera de expresarse. \| \| CIS23 \| sm \| 23 \| In social situations, I have the ability to alter my behavior if I feel that something else is called for \| En situaciones sociales tengo la habilidad de modificar mi comportamiento si siento que algo lo requiere. \| \| CIS25 \| sm \| 25 \| I have found that I can adjust my behavior to meet the requirements of any situation I find myself in. \| He descubierto que puedo ajustar mi comportamiento ante cualquier situación en la que me encuentre. \| \| CIS27  Reversed \| sm \| 27 \| Even when it might be to my advantage, I have a difficulty putting up a good front. \| Aun cuando podría ser positivo para mi, tengo dificultades para poner buena cara. \| \| CIS28 \| so \| 28 \| I can usually tell when others consider a joke to be in bad taste, even though they may laugh convincingly \| Normalmente puedo decir cuando otros consideran que hago una broma de mal gusto, a pesar de que pueden reírse de manera convincente. \| \| CIS30 \| sm \| 30 \| When I feel that the image I am portraying isn't working, I can readily change it to something that does. \| Cuando siento que la imagen que estoy mostrando no funciona, puedo fácilmente intentar cambiar a otra que si funcione. \| \| CIS31 \| sm \| 31 \| I have the ability to control the way I come across to people, depending on the impression I wish to give them \| Tengo la habilidad de controlar la manera en la que abordo a la gente, dependiendo de la impresión que quiero que tengan de mi. \| |
| --- | --- | --- | --- | --- | --- | --- | --- | --- | --- | --- | --- | --- | --- | --- | --- | --- | --- | --- | --- | --- | --- | --- | --- | --- | --- | --- | --- | --- | --- | --- | --- | --- | --- | --- | --- | --- | --- | --- | --- | --- | --- | --- | --- | --- | --- | --- | --- | --- | --- | --- | --- | --- | --- | --- | --- | --- | --- | --- | --- | --- | --- | --- | --- | --- | --- | --- | --- | --- | --- | --- |
|  |
|  |

**Tromso Social Intelligence Scale:**

**3 subscales: Social Information Processing, Social Skills, and Social Awareness**

Silvera, D., Martinussen, M. and Dahl, T. I. (2001), The Tromsø Social Intelligence Scale, a self-report measure of social intelligence. Scandinavian Journal of Psychology, 42: 313–319. doi: 10.1111/1467-9450.00242

[Measures spain\Tromso\The Tromsø Social Intelligence Scale, a self-report measure of social intelligence - Silvera - 2001 - Scandinavian Journal of Psychology - Wiley Online Library.pdf](file:///C:\Users\atajz\Documents\Dropbox\SI%20Carmen,%20Alex%20and%20Eva\Measures%20spain\Tromso\The%20Tromsø%20Social%20Intelligence%20Scale,%20a%20self-report%20measure%20of%20social%20intelligence%20-%20Silvera%20-%202001%20-%20Scandinavian%20Journal%20of%20Psychology%20-%20Wiley%20Online%20Library.pdf)

[Measures spain\Tromso\Tromso for adolescents.pdf](file:///C:\Users\atajz\Documents\Dropbox\SI%20Carmen,%20Alex%20and%20Eva\Measures%20spain\Tromso\Tromso%20for%20adolescents.pdf)

Scale in English Scale in Spanish

1. Not like me at all! 1 NO! En absoluto es como yo

2 2

3. Somewhat like me 3 Algo parecido a mi

4. 4

5. Very much like me 5 SI! Muy parecido a mi

**Total SI=SITOT**

**Subscale: Social Skills (siss) Total siss=SOCIALSKILLS_si**

| **Variable** | **Item in English** | **Item in Spanish** |
| --- | --- | --- |
| CIS4  Reverse | I often feel uncertain around new people who I don’t know | A menudo me siento inseguro entre nuevas personas que no conozco. |
| CIS29 | I fit in easily in social situations and meeting people for the first time | Encajo bien en nuevas situaciones sociales y en conocer a la gente por primera vez. |
| CIS34 | I am good at entering new situations and meeting people for the first time | Soy bueno para afrontar nuevas situaciones sociales y para conocer gente por primera vez. |
| CIS18  Reverse | I have a hard time getting along with other people | Me resulta difícil llevarme bien con otras personas. |
| CIS19  Reverse | It takes a long time for me to get to know others well | Me lleva bastante tiempo llegar a conocer bien a otras personas. |
| CIS14 | I am good at getting on good terms with new people | Tengo habilidad para quedar bien con personas desconocidas |
| CIS32  Reverse | I frequently have problems finding good conversation topics | Frecuentemente tengo problemas para encontrar buenos temas sobre los que conversar. |

**Subscale: Social Awareness (SISA) Total sisa=AWARENESS_SI**

| **Variable** | **Item** | **Spanish** |
| --- | --- | --- |
| CIS6  Reverse | I have often hurt others without realizing it. | Algunas veces hago daño a los demás sin darme cuenta. |
| CIS8  Reverse | I find people are unpredictable | Encuentro a la gente impredecible. |
| CIS15  Reverse | I often feel that it is difficult to understand others’ choices | A menudo siento que es difícil entender las decisiones de los otros. |
| CIS20  Reverse | It seems as though people are often angry or irritated with me when I say what I think | Parece que la gente a menudo está enojada conmigo cuando digo lo que pienso. |
| CIS24  Reverse | People often surprise me with the things they do | A menudo la gente me sorprende con las cosas que hace. |
| CIS33  Reverse | Other people become angry with me without being able to explain why | Otras personas pueden enfadarse conmigo sin que yo pueda llegar a saber por qué. |
| CIS35  Reverse | I am often surprised by others’ reactions to what I do | A menudo me sorprendo ante las reacciones de los demás ante lo que hago. |

**Subscale: Social Information Processing (sisi) Total sisi=Processing_SI**

| **Variable** | **Item** | **Spanish** |
| --- | --- | --- |
| CIS12 | I can predict other peoples’ behavior | Puedo predecir el comportamiento de los demás. |
| CIS21 | I know how my actions will make others feel | Sé como mis acciones harán sentir a los demás. |
| CIS7 | I understand other people’s feelings | Comprendo los sentimientos de los demás. |
| CIS26 | I understand other’s wishes | Comprendo los deseos de otros. |
| CIS22 | I can often understand what others are trying to accomplish without the need for them to say anything | A menudo puedo entender lo que los otros están tratando de lograr sin la necesidad de que me digan nada. (This translation says “me” rather than “them” |
| CIS17 | I can predict how others will react to my behavior | Puedo predecir como reaccionarán los demás ante mi comportamiento |
| CIS11 | I can often understand what others really mean through their expression, body language, etc. | A menudo puedo entender lo que otros quieren decir a través de su expresión, lenguaje corporal…etc. |

**This following table is a combined table of self-monitoring scale and Tromso scale. Use it to sort etc. It is currently sorted by items in Questionnaire (Survey)**

| **Variable** | **Subscale** | **Survey** | **Item in English** | **Item in Spanish** | |  |
| --- | --- | --- | --- | --- | --- | --- |
| CIS2 | so | 2 | In conversations, I am sensitive to even the slightest change in the facial expressions of the person I’m conversing with | En una conversación soy sensible incluso al mínimo cambio en la expresión de la cara de la persona con la que estoy conversando. | |  |
| CIS3 | so | 3 | My powers of intuition are quite good when it comes to understanding other’s emotions and motives. | Mis poderes de intuición son muy buenos a la hora de entender las emociones y las motivaciones de los demás. | |  |
| CIS4  Reverse | Siss | 4 | I often feel uncertain around new people who I don’t know | A menudo me siento inseguro entre nuevas personas que no conozco. | |  |
| CIS5 | so | 5 | I am often able to read people’s true emotions correctly through their eyes | A menudo soy capaz de leer las verdaderas emociones de las personas a través de sus ojos. | |  |
| CIS6  Reverse | sisa | 6 | I have often hurt others without realizing it. | Algunas veces hago daño a los demás sin darme cuenta. | | |
| CIS7 | sisi | 7 | I understand other people’s feelings | Comprendo los sentimientos de los demás. | | |
| CIS8  Reverse | sisa | 8 | I find people are unpredictable | Encuentro a la gente impredecible. | | |
| CIS9 | so | 9 | I can usually tell when I’ve said something inappropriate by reading it in the listener’s eyes | Puedo saber cuando he dicho algo inapropiado leyendo en los ojos de la otra persona. | | |
| CIS10  Reverse | sm | 10 | I have trouble changing my behavior to suit different people and different situations | Tengo problemas para cambiar mi comportamiento y adaptarme a diferentes personas en diferentes situaciones. | | |
| CIS11 | sisi | 11 | I can often understand what others really mean through their expression, body language, etc. | A menudo puedo entender lo que otros quieren decir a través de su expresión, lenguaje corporal…etc. | | |
| CIS12 | sisi | 12 | I can predict other peoples’ behavior | Puedo predecir el comportamiento de los demás. | | |
| CIS13 | sm | 13 | Once I know what the situation calls for, it’s easy for me to regulate my actions accordingly | Una vez que sé lo que la situación requiere, es fácil para mi regular mis acciones en consecuencia. | | |
| CIS14 | siss | 14 | I am good at getting on good terms with new people | Tengo habilidad para quedar bien con personas desconocidas | | |
| CIS15  Reverse | sisa | 15 | I often feel that it is difficult to understand others’ choices | A menudo siento que es difícil entender las decisiones de los otros. | | |
| CIS16 | so | 16 | If someone is lying to me, I usually know it at once from that person’s manner of expression | Si alguien me esta mintiendo, normalmente lo se desde el principio por su manera de expresarse. | |  |
| CIS17 | sisi | 17 | I can predict how others will react to my behavior | Puedo predecir como reaccionarán los demás ante mi comportamiento | | |
| CIS18  Reverse | siss | 18 | I have a hard time getting along with other people | Me resulta difícil llevarme bien con otras personas. | |  |
| CIS19  Reverse | siss | 19 | It takes a long time for me to get to know others well | Me lleva bastante tiempo llegar a conocer bien a otras personas. | |  |
| CIS20  Reverse | sisa | 20 | It seems as though people are often angry or irritated with me when I say what I think | Parece que la gente a menudo está enojada conmigo cuando digo lo que pienso. |  |  |
| CIS21 | sisi | 21 | I know how my actions will make others feel | Sé como mis acciones harán sentir a los demás. |  |  |
| CIS22 | sisi | 22 | I can often understand what others are trying to accomplish without the need for them to say anything | A menudo puedo entender lo que los otros están tratando de lograr sin la necesidad de que me digan nada. (This translation says “me” rather than “them” |  |  |
| CIS23 | sm | 23 | In social situations, I have the ability to alter my behavior if I feel that something else is called for | En situaciones sociales tengo la habilidad de modificar mi comportamiento si siento que algo lo requiere. | | |
| CIS24  Reverse | sisa | 24 | People often surprise me with the things they do | A menudo la gente me sorprende con las cosas que hace. |  |  |
| Cis25 | sm | 25 | I have found that I can adjust my behavior to meet the requirements of any situation I find myself in. | He descubierto que puedo ajustar mi comportamiento ante cualquier situación en la que me encuentre. | |  |
| CIS26 | sisi | 26 | I understand other’s wishes | Comprendo los deseos de otros. |  |  |
| CIS27  Reverse | sm | 27 | Even when it might be to my advantage, I have a difficulty putting up a good front. | Aun cuando podría ser positivo para mi, tengo dificultades para poner buena cara. | |  |
| Cis28 | so | 28 | I can usually tell when others consider a joke to be in bad taste, even though they may laugh convincingly | Normalmente puedo decir cuando otros consideran que hago una broma de mal gusto, a pesar de que pueden reírse de manera convincente. | |  |
| CIS29 | siss | 29 | I fit in easily in social situations and meeting people for the first time | Encajo bien en nuevas situaciones sociales y en conocer a la gente por primera vez. | |  |
| Cis30 | sm | 30 | When I feel that the image I am portraying isn't working, I can readily change it to something that does. | Cuando siento que la imagen que estoy mostrando no funciona, puedo fácilmente intentar cambiar a otra que si funcione. | |  |
| Cis31 | sm | 31 | I have the ability to control the way I come across to people, depending on the impression I wish to give them | Tengo la habilidad de controlar la manera en la que abordo a la gente, dependiendo de la impresión que quiero que tengan de mi. | |  |
| CIS32  Reverse | siss | 32 | I frequently have problems finding good conversation topics | Frecuentemente tengo problemas para encontrar buenos temas sobre los que conversar. | |  |
| CIS33  Reverse | sisa | 33 | Other people become angry with me without being able to explain why | Otras personas pueden enfadarse conmigo sin que yo pueda llegar a saber por qué. |  |  |
| CIS34 | siss | 34 | I am good at entering new situations and meeting people for the first time | Soy bueno para afrontar nuevas situaciones sociales y para conocer gente por primera vez. | |  |
| CIS35  Reverse | sisa | 35 | I am often surprised by others’ reactions to what I do | A menudo me sorprendo ante las reacciones de los demás ante lo que hago. |  |  |

**Self Efficacy Scale**

I don’t believe Nancy ever published anything about this scale although we used it at Metro Arts. What does CAUG mean?

Gonzales, N. (2013). The measurement of social efficacy and anxiety in social relations in adolescents and young adults. Unpublished manuscript. See [Measures spain\Self Efficacy Scale (Gonzales)\General Self-efficacy scale.png](file:///C:\Users\atajz\Documents\Dropbox\SI%20Carmen,%20Alex%20and%20Eva\Measures%20spain\Self%20Efficacy%20Scale%20(Gonzales)\General%20Self-efficacy%20scale.png)

Scale in English Scale in Spanish

1 Not at all true 1 Incorrecto

2 Hardly true 2 Apenas cierto

3 Moderately true 3 Mas bien cierto

4 Exactly true 4 Cierto

**Totals: autoeficacia_PRE and autoeficacia_POST**

| **Variable** | **Item in English** | **Item in Spanish** |
| --- | --- | --- |
| CAUG1 | If someone opposes me, I can find he means and ways to get what I want. | Puedo encontrar la manera de obtener lo que quiero aunque alguien se me oponga. |
| CAUG10 | When I am confronted with a problem, I can usually find several solutions. | Al tener que hacer frente a un problema, generalmente se me ocurren varias alternativas de cómo resolverlo. |
| CAUG2 | I can solve most problems if I invest the necessary effort. | Puedo resolver problemas difíciles si me esfuerzo lo suficiente. |
| CAUG3 | It is easy for me to stick to my aims and accomplish my goals. | Me es fácil persistir en lo que me he propuesto hasta llegar a alcanzar mis metas. |
| CAUG4 | I am confident that I could deal efficiently with unexpected events. | Tengo confianza en que podría manejar eficazmente acontecimientos inesperados. |
| CAUG5 | Thanks to my resourcefulness. I know how to handle unforeseen situations. | Gracias a mis cualidades y recursos puedo superar situaciones imprevistas. |
| CAUG6 | I can remain calm when facing difficulties because I can rely on m coping abilities. | Cuando me encuentro en dificultades puedo permanecer tranquilo/a porque cuento con las habilidades necesarias para manejar situaciones difíciles. |
| CAUG7 | I can usually handle whatever comes my way. | Venga lo que venga, por lo general soy capaz de manejarlo. |
| CAUG8 | I can always manage to solve difficult problems if I try hard enough | Puedo resolver la mayoría de los problemas si me esfuerzo lo necesario. |
| CAUG9 | If I am in trouble, I can usually think of a solution. | Si me encuentro en una situación difícil, generalmente se me ocurre qué debo hacer. |

**Interpersonal Reactivity Index (IRI)**

The Interpersonal Reactivity Index (Davis, 1980, 1983) is a measure of dispositional empathy that takes as its starting point the notion that empathy consists of a set of separate but related constructs. The instrument contains four seven-item subscales, each tapping a separate facet of empathy. The perspective taking (PT) scale measures the reported tendency to spontaneously adopt the psychological point of view of others in everyday life ("I sometimes try to understand my friends better by imagining how things look from their perspective"). The empathic concern (EC) scale assesses the tendency to experience feelings of sympathy and compassion for unfortunate others ("I often have tender, concerned feelings for people less fortunate than me"). The personal distress (PD) scale taps the tendency to experience distress and discomfort in response to extreme distress in others ("Being in a tense emotional situation scares me"). The fantasy (FS) scale measures the tendency to imaginatively transpose oneself into fictional situations ("When I am reading an interesting story or novel, I imagine how I would feel if the events in the story were happening to me").

[Measures spain\IRI\Fetzer IRI.pdf](file:///C:\Users\atajz\Documents\Dropbox\SI%20Carmen,%20Alex%20and%20Eva\Measures%20spain\IRI\Fetzer%20IRI.pdf)

- **Davis, M. H.** (1980). A multidimensional approach to individual differences in empathy. *JSAS Catalog of Selected Documents in Psychology, 10*, 85.
- **Davis, M. H.** (1983). Measuring individual differences in empathy: Evidence for a multidimensional approach. *Journal of Personality and Social Psychology, 44*, 113-126.

Instrucciones: Las siguientes frases se refieren a tus pensamientos y sentimientos en una variedad de situaciones. Para cada cuestión indica cómo te describe eligiendo la puntuación de 1 a 5 (1= no me describe bien, 2 = me describe un poco, 3 = me describe bien, 4 = me describe muy bien y 5 = me describe bastante bien). Cuando hayas elegido tu respuesta, marca con una cruz la casilla correspondiente. Lee cada frase cuidadosamente antes de responder.

Scale in English Scale in Spanish

1 Does not describe me at all 1 No me describe bien

2 Describes me slightly 2 Me describe un poco

3 Describes me somewhat 3 Me describe bien

4 Describes me moderately well 4 Me describe bastante

5 Describes me extremely well 5 Me describe muy bien

Subscale totals

IRI_EC (Emotional concern)

IRI_TP (Perspective taking)

| Variable | Subscale | Item English | Item Spanish |
| --- | --- | --- | --- |
| IRI1 | EC | I often have tender, concerned feelings for people less fortunate than me. | A menudo tengo sentimientos tiernos y de preocupación hacia la gente menos afortunada que yo. |
| IRI2  Reverse | PT | I sometimes find it difficult to see things from the "other guy's" point of view. | A menudo encuentro difícil ver las cosas desde el punto de vista de otra persona. |
| IRI3  Reverse | EC | Sometimes I don't feel very sorry for other people when they are having problems. | A veces no me siento muy preocupado por los problemas de otras personas. |
| IRI4 | PT | I try to look at everybody's side of a disagreement before I make a decision. | Intento tener en cuenta cada una de las partes (opiniones) en un conflicto antes de tomar una decisión. |
| IRI5 | EC | When I see someone being taken advantage of, I feel kind of protective towards them. | Cuando veo que a alguien se le toma el pelo tiendo a protegerlo. |
| IRI6 | PT | I sometimes try to understand my friends better by imagining how things look from their perspective. | A menudo intento comprender a mis amigos imaginándome cómo ven ellos las cosas (poniéndome en su lugar). |
| IRI7  Reverse | EC (PD) | When I see someone get hurt, I tend to remain calm. | Cuando veo a alguien herido tiendo a permanecer calmado. |
| IRI8  Reverse | PT | If I am sure that the reason I have something I do not waste time listening to arguments of others. | Si estoy seguro que tengo la razón en algo no pierdo tiempo escuchando los argumentos de los demás. |
| IRI9 | EC | I am often quite emotionally affected by what I see occurring. | A menudo estoy bastante afectado emocionalmente por cosas que veo que ocurren. |
| IRI10 | PT | I think that there are two sides to every issue and try to take both sides into account. | Pienso que hay dos partes para cada cuestión e intento tener en cuenta ambas partes. |
| IRI11 | EC | I would describe as a very sensitive person. | Me describiría como una persona bastante sensible. |
| IRI12 | PT | When I am upset with someone normally I try to put me in his place for a moment. | Cuando estoy disgustado con alguien normalmente intento ponerme en su lugar por un momento. |
| IRI13 | PT | Before you criticize someone attempt to imagine how I would feel if you were in place. | Antes de criticar a alguien intento imaginar cómo me sentiría si estuviera en su lugar. |

**Trait Meta-Mood Scale (TMMS24)** is a measure of perceived emotional intelligence: that is, individuals’ beliefs about their own emotional abilities.. The original version is a 48-item questionnaire, although the use of abridged versions such as the 30-item and 24-item ones is recommended.

*Emotion, Disclosure, and Health*, pp 152-154, Copyright @1995 by the American Psychological Association, Adapted with permission. Salovey, P Mayer, J.D., Goldman, S., Turvey, C. & Palfai, T. (1995). Emotional attention clarity and repair: Exploring emotional intelligence using the trait Meta-Mood Scale. In J.W. Pennebaker (Ed.) *Emotion, disclosure, and health* (pp125-154). Washington DC: American Psychological Association.

Fernandez-Berrocal, P., Extremera, N. y Ramos, N. (2004). Validity and reliability of the Spanish modified version of the Trait Meta-Mood Scale. Psychological Reports, 94, 751-755.

Instructions in Spanish (No instructions in actual survey)

A continuation encontrara algunas afirmaciones sobre sus emotions y sentimientos. Lea atentamente cada frase y indigue por favor el gado de acuerdo o desacuerdo con respedto a las mismas. Senales con una “X” in respuesta que mas se aproxime a sus preferencias. No hay respuestas correctas o incorrectas, ni buenas or malas. No emplee mucho tiempo en cada respuesta.

Instructions in English. Please read each statement and decide whether or not you agree with it.

Scale in English Scale in Spanish

1 Strongly disagree 1 Nada de acuerdo

2 Somewhat disagree 2 Algo de acuerdo

3 Neither agree nor disagree 3 Bastante de acuerdo

4 Somewhat agree 4 Muy de acuerdo

5 Strongly agree 5 Totalmente de acuerdo

Total scale: INTELIG_EMOC

**Subscales**

A=ATENICON_TMMS: (Attention) the degree to which the individual observes and thinks about their feelings (e.g., “I pay a lot of attention to how I feel”)

C=CLARIDAD_TMMS: (Clarity)_The understanding of one’s emotional states (e.g., “I am usually very clear about my feelings”)

R=REPARACION_TMMS: (Repair) The ability to regulate one’s feelings (e.g., “When I become upset, I remind myself of all the pleasures in life”)

The English version of the TMMS is completely different. Has many reverse items.

| Variable | Subscale | Item in Spanish | From English (from literature) |
| --- | --- | --- | --- |
| TMMS24_1 | Attention  (A) | Presto mucha atención a los sentimientos. | I pay much attention to my feelings. |
| TMMS24_2 | Attention  (A) | Normalmente me preocupo mucho por lo que siento. | Usually I care much about what I am feeling. |
| TMMS24_3 | Attention  (A) | Normalmente dedico tiempo a pensar en mis emociones. | I usually spend time thinking about my emotions. |
| TMMS24_4 | Attention  (A) | Pienso que merece la pena prestar atención a mis emociones y estado de ánimo. | I think it's worth paying attention to my emotions and mood. |
| TMMS24_5 | Attention  (A) | Dejo que mis sentimientos afecten a mis pensamientos. | I let my emotions interfere with my thoughts |
| TMMS24_6 | Attention  (A) | Pienso en mi estado de ánimo constantemente. | I think about my mood constantly |
| TMMS24_7 | Attention  (A) | A menudo pienso en mis sentimientos. | I often think about my feelings. |
| TMMS24_8 | Attention  (A) | Presto mucha attention a como me siento. | I pay a lot of attention to how I feel. |
| TMMS24_9 | Clarity (C) | Tengo claros mis sentimientos. | I am clear about my feelings |
| TMMS24_10 | Clarity(C) | Frecuentemente puedo definer mis sentimientos. | I am rarely confused about how I feel |
| TMMS24_11 | Clarity(C) | Casi siempre se como me siento | I almost always know exactly how I am feeling (I usually know my feelings about a matter) |
| TMMS24_12 | Clarity (C) | Normalmente conozco mis sentimientos sobre las personas. | I usually know my feelings about people. (I can make sense out of my feelings) |
| TMMS24_13 | Clarity(C) | A menudo me doy cuenta de mis sentimientos en diferentes situaciones, | I am often aware of my feelings in different situations (on a matter). |
| TMMS24_14 | Clarity (C) | Siempre puedo decir cómo me siento. | I can always tell how I feel. |
| TMMS24_15 | Clarity(C) | A veces puedo decir cuáles son mis emociones. | Sometimes I can tell what my feelings are. |
| TMMS24_16 | Clarity (C) | Puedo llegar a comprender mis sentimientos. | I almost always know exactly how I am feeling. (I can come to understand my feelings.) |
| TMMS24_17 | Repair (R) | Aunque a veces me siento triste, suelo tener una vision optimista | Although I am sometimes sad, I have a mostly optimistic outlook |
| TMMS24_18 | Repair(R) | Aunque me sienta mal, procuro pensar en cosas agradables. | I try to think good thoughts no matter how badly I feel. |
| TMMS24_19 | Repair(R) | Cuando estoy triste, pienso en todos los placeres de la vida. | When I become upset I remind myself of all the pleasures in life. |
| TMMS24_20 | Repair (R) | Intento tener pensamientos positivos aunque me sienta mal. | I try to think good thoughts no matter how badly I feel. |
| TMMS24_21 | Repair (R) | Si doy demasiadas vueltas a las cosas, complicándolas, trato de calmarme. | If I find myself getting mad, I try to calm myself down. |
| TMMS24_22 | Repair (R) | Me preocupo por tener un buen estado de ánimo. | I worry about being in too good a mood. (This is a strange one. Does anyone know why it isn’t reversed?) |
| TMMS24_23 | Repair (R) | Tengo mucha energia cuando me siento feliz. | I have a lot of energy when I am happy. |
| TMMS24_24 | Repair (R) | Cuando estoy enfadado intento cambiar mi estado de ánimo. | When I’m angry, I usually try to change my mood, |
